# Variation in insulin response to oral sugar test in a cohort of horses throughout the year and evaluation of risk factors for insulin dysregulation

**DOI:** 10.1111/evj.13529

**Published:** 2021-11-08

**Authors:** Ninja P. Karikoski, Justin R. Box, Anna K. Mykkänen, Veikko V. Kotiranta, Marja R. Raekallio

**Affiliations:** ^1^ Department of Equine and Small Animal Sciences Faculty of Veterinary Medicine University of Helsinki Helsinki Finland

**Keywords:** endocrine, horse, insulin dysregulation, seasonal

## Abstract

**Background:**

The oral sugar test (OST) is commonly used to diagnose insulin dysregulation (ID) and equine metabolic syndrome; however, possible seasonal changes in OST results have not been evaluated.

**Objective:**

To determine the possible variation in insulin response to OST throughout the year and risk factors associated with maximum insulin concentration (InsMax) and ID.

**Study design:**

Prospective, longitudinal cohort study.

**Methods:**

The OST was performed on 29 Finnhorses every other month six times. Serum total adiponectin concentration and phenotypic variables related to obesity were also measured. Changes in InsMax, adiponectin, scale weight, body condition score, cresty neck score (CNS), and fasting glucose concentration were assessed. Risk factor analyses were performed on InsMax and ID status, and ID groups were compared with each other.

**Results:**

Fourteen horses were categorised with non‐ID each time and 15 as having ID at least once during the follow‐up period. The ID status of 12 horses varied throughout the year, but neither the insulin variables measured during the OST nor adiponectin expressed significant seasonal variation. Increasing age and CNS, and decreasing adiponectin were observed as risk factors for a high InsMax after OST. The risk of ID was higher in horses with no exercise compared to horses with exercise (OR 7.6, 95% CI 1.2‐49.3, *P* = .03). Horses with ID had lower serum adiponectin concentrations, longer neck circumference and larger height than horses in the non‐ID group.

**Main limitations:**

The environmental conditions (feeding, exercise) were not constant for all horses throughout the study and only one breed was used.

**Conclusions:**

Neither OST results nor adiponectin varies with season; however, there were a substantial number of horses with variable ID status throughout the year, in which repeated OSTs may be beneficial. Lack of exercise was a risk factor for ID.

## 1 INTRODUCTION

Horses have variation in their metabolic activity throughout the year. In wild Przewalski horses, seasonal changes in heart rate, body temperature, locomotor activity, and heat increment were shown to occur independent of nutrient availability.^1^ Decreased metabolic activity was present during winter, and this was later also observed in Shetland ponies, indicating that domesticated horses may still have the ability to adjust their metabolism according to environmental conditions.^2^ However, the exact mechanisms on how this adjustment occurs in horses (eg at the hormonal level) are not well described.

The activity of the hypothalamic‐pituitary‐adrenal axis in horses increases in autumn when the daylight decreases, possibly preparing the animal for the winter. This increased activity can be observed by increased plasma concentrations of pro‐opiomelanocortin‐derived hormones such as adrenocorticotropic hormone (ACTH)^3^, ^4^, ^5^, ^6^, ^7^, ^8^, ^9^, ^10^ and α‐melanocyte‐stimulating hormone (α‐MSH).^4^, ^5^, ^6^, ^7^, ^8^ These hormones have several functions in maintaining body homeostasis including regulation of energy metabolism. The main role of ACTH is stimulation of adrenal cortisol release, whereas α‐MSH is thought to contribute to the development of obesity. Both hormones can also affect insulin regulation^11^, ^12^; however, studies evaluating the seasonality of equine blood insulin concentrations have yielded inconclusive or conflicting results. Some studies have revealed significant differences between seasons,^4^, ^7^, ^13^, ^14^, ^15^, ^16^ while other studies have shown contrasting results.^5^, ^17^, ^18^

Equine metabolic syndrome (EMS) is characterised by obesity (either generalised or regional), insulin dysregulation (ID) and a predisposition to laminitis. Dynamic oral sugar tolerance tests (OST) are often used in EMS diagnosis.^19^ However, adipokine concentrations, such as adiponectin, may also be used as a diagnostic tool.^20^, ^21^, ^22^ The seasonality of OST response has not been previously evaluated. Additionally, while monthly fluctuations in adiponectin have been observed in horses, a seasonal pattern has not been established.^16^

This prospective study was undertaken to determine the variations in insulin response to OST, adiponectin, and phenotypic markers of obesity in a cohort of Finnhorses throughout the year. Additionally, we sought to analyse potential risk factors associated with increased insulin response and ID status. We hypothesised that these metabolic hormones and the phenotypic markers of obesity would follow a seasonal pattern.

## 2 MATERIALS AND METHODS

In this longitudinal study, experiments were performed every other month for a total of six times, beginning in June 2018 and ending in April 2019. The median time between the experiments was 63 days (range 55‐70 days).

### 2.1 Animals

A convenience sample of 29 Finnhorses from 3 to 19 years old (mean ± SD 11.8 ± 3.4 years) owned by the Natural Resources Institute and the Ypäjä Equine College were used for this study (all available non‐pregnant Finnhorses over 3 years of age in these institutes). All animals were housed at the same facility (Ypäjä, latitude 60.8°N, longitude 23.3°W). Initially, there were 10 geldings and 19 non‐pregnant mares. The mean weight (n = 29) recorded on scales of the horses at the beginning of the experiment was 579 ± 46 kg (range 509‐664 kg), and mean height was 157 ± 5 cm (range 146‐168 cm). A physical examination, serum biochemistry panel (Konelab 30; ThermoFisher Scientific), complete blood count (ADVIA 212io haematology analyser), and fibrinogen concentration (heat precipitation method)^23^ were performed on all horses on each test date.

### 2.2 Physical measurements

The following physical measurements were obtained on each test day with an equine weight tape (Virbac Animal Health): weight, heart‐girth, widest part of the abdomen, and neck circumference (midpoint of the neck). Scale weight was measured within 1 week of each test day. Additionally, body condition score (BCS)^24^ and cresty neck score (CNS)^25^ were assessed by one of two trained veterinarians (NPK, JRB).

### 2.3 Feeding and exercise

Feeding and exercise were kept as constant as possible throughout the year. All horses were fed hay or haylage (median [range]: 8 kg/d [7‐13.3 kg/d]) and small amounts of oats, concentrates, or both (median [range]: 0.64 kg/d [0.1‐2.4 kg/d]) from October 2018 to May 2019. From June 2018 to September 2018, 23/29 horses were kept on pasture from 1 to 4 months (median of all the horses 1 month, range 0‐4 months). For statistical analysis, feeding was recorded every month as pasture or inside feeding.

The exercise level was constant from October 2018 to May 2019. Seven horses did not exercise at all, but the remaining 22/29 horses were used for riding and driving school lessons (intensity from light to moderate) and exercised on average 1.2 hours per day (median [range]: 1.3 h/d [0.6‐1.3 hours]). From June 2018 to September 2018, the exercise level changed for 6/22 horses when they were turned on pasture (from being exercised to rest). For statistical analysis, exercise was recorded as exercise or no exercise.

### 2.4 Blood samples

Fasting blood glucose (BG) concentration was determined using lithium‐heparin blood (Vacuette LH; Greiner Bio‐One) immediately after sampling using a handheld glucometer (AlphaTRAK II; Zoetis) calibrated for horses.

Blood for plasma ACTH concentration measurement was collected in 6‐mL EDTA tubes (Vacuette K2EDTA; Greiner Bio‐One) and kept cool until centrifugation (within 8 hours). The separated plasma was frozen and stored for a maximum of 44 days (median [range]: 9.5 days [3‐44 days]) at −80°C until sent on dry ice to the laboratory. Analysis of ACTH samples was performed using a chemiluminescent immunoassay (Immulite 1000; Rainbow Equine Hospital). The seasonally adjusted ACTH cut‐off values (Northern hemisphere) used for this study were 34.9 pg/mL (February‐April), 44.5 pg/mL (May‐July), 89.4 pg/mL (August‐October), and 35.2 pg/mL for horses sampled in December.^26^

Blood for serum insulin and total adiponectin concentration analysis was collected in 6‐mL serum tubes (Vacuette, Z serum clot activator; Greiner Bio‐One) before syrup administration and for insulin also at 60, 90, and 120 (T60‐120) minutes thereafter. Samples were allowed to clot at room temperature for at least 1 hour, centrifuged (within 8 hours), and the separated serum was stored at −80°C until sent on dry ice to the laboratory. Insulin concentrations were measured using a chemiluminescent immunoassay (Immulite 1000) with a reportable range of 2‐300 µIU/mL. Adiponectin was measured by an immunoturbidimetric assay (Randox Reagents) validated for horses.^27^

### 2.5 Oral sugar test

The evening before OSTs, the horses were confined to stalls and allowed to have a slice of dry hay (1‐2 kg) no later than 22:00. No grain or additional hay was allowed until after the OST was completed. All horses had access to water throughout the entire experiment. All OSTs were started in the morning between 06:00 and 09:00. Horses were given 0.45 mL/kg body weight of corn syrup (Karo Light; ACH Food Companies Inc.) PO via 100‐mL dosing syringes.

Horses were categorised as having ID based on insulin concentrations ≥20 µIU/mL at T0 or ≥40 µIU/mL at either T60, T90, T120, or all timepoints.^19^

### 2.6 Data analysis

Data analysis was performed using statistical software SAS (SAS System for Windows, version 9.4). The maximum insulin concentration (InsMax) detected at T60, T90, or T120 for each horse on each testing day was determined and used as a continuous variable for statistical analysis and modelling. Insulin values >300 µIU/mL were reported as 300 µIU/mL. The area under the time versus insulin concentration curve (AUC_ins_) was calculated for the insulin response (T0‐T120) using the trapezoidal method. The normality assumptions for all repeated measures analysis of variance (RM ANOVA) and repeated measures analysis of covariance (RM ANCOVA) models were assessed with Kolmogorov‐Smirnov tests. For InsMax and adiponectin, a logarithmic transformation was used to normalise the distribution. For a few variables (neck circumference, height, age, AUC_ins_), the normality assumption could not be verified with the test. After investigating these variables graphically and with skewness and kurtosis measures, normality was seen as adequate for analysis. For AUC_ins_, however, a non‐parametric analysis was considered more reliable.

#### 2.6.1 Changes throughout the year

Changes in InsMax, AUC_ins_, adiponectin, scale weight, BCS, CNS, and fasting BG throughout the year were investigated. Due to the small number of observations at some timepoints, BCS classes 5 and 6 and 8 and 9 were combined for analysis. For InsMax, adiponectin, scale weight, and fasting BG, a RM ANOVA model was fitted that included the response, fixed effect of time point, and random effect of horse. For BCS and CNS, a mixed effects cumulative logistic regression model (modelling the odds for higher scores) with fixed effect of timepoint and random effect of horse was fitted. For AUC_ins_, changes were evaluated with a Friedman test, as the distribution of AUC was skewed with many outliers.

In RM ANOVA models, within‐timepoint values for each response with 95% confidence intervals (CI) and *P*‐values were estimated from the same model with contrasts. In the cumulative models, odds ratios (OR) with 95% CI and *P*‐values for timepoint comparisons were computed with contrasts from the same model. *P*‐values ≤.05 were considered statistically significant.

#### 2.6.2 Risk factor analyses

The following variables were considered as potential risk factors for InsMax and ID and were used in risk factor analyses: age, sex, timepoint (month), adiponectin, scale weight, BCS, heart‐girth, widest part of the abdomen, CNS, neck circumference, feeding, and exercise. For risk factor analyses of InsMax, RM ANCOVA models were used to assess the associations between the response, covariates, and timepoint. The analyses were performed in three stages. First, each covariate was modelled separately with the response (univariate analysis). In this stage, in addition to the response, the model included the covariate, timepoint, and their interaction as fixed effects, and horse as a random effect. Second, the covariates were classified into three subgroups: demographic (age, sex), phenotypic and biochemical values (adiponectin, BCS, CNS, girth, mid‐neck circumference, scale weight, widest part of the abdomen), and external conditions (exercise, feeding). The covariates that were significant in the first stage were included in the models with timepoint. None of the interactions of covariate and timepoint were significant in the first stage and therefore no interactions were included in the subgroup models. A final model of significant subgroup covariates and timepoint was fitted. Random effect of horse was included in the models in all stages. In the stage one and three models, differences in response between groups (categorical covariates) or increase of one unit (continuous or ordinal covariate) with 95% CI and *P*‐values were estimated with contrasts from the same model.

The risk factor analyses for ID were performed in a similar manner as described for InsMax; however, a mixed effects logistic regression model was used to model the odds for occurrence of ID. In all models, comparisons between groups (categorical covariates) or increase of one unit (continuous or ordinal covariate) with 95% CI and *P*‐values were estimated as OR with contrasts from the same model. *P*‐values ≤.05 were considered statistically significant.

#### 2.6.3 Insulin dysregulation group comparison

Differences in adiponectin, fasting BG, widest part of the abdomen, neck circumference, height, heart‐girth, age, and scale weight between two groups were investigated using RM ANOVA. The groups were defined as ID at least once during the follow‐up period (ID) or never ID (non‐ID). Differences in BCS and CNS between the groups were investigated with mixed effects cumulative logistic regression models. Each analysis model included the response, ID group, timepoint, and interaction of timepoint and ID group as fixed effects, and horse as a random effect. Due to the small number of observations in some classes, for analyses of BCS and CNS, BCS classes 5 and 6, as well as 8 and 9 were combined, and CNS classes 1 and 2 were combined.

## 3 RESULTS

### 3.1 OST

Oral sugar test was performed altogether 171 times during 1 year. Maximum insulin concentration occurred at T0 in 1/171 OST, at T60 in 61/171 OSTs, at T90 in 46/171 OSTs and at T120 in 57/171 OSTs. In six OSTs the InsMax timing could not be determined either due to the horse not responding to oral sugar (all the insulin values under the detection limit; n = 3, always a different horse) or due to all the postprandial values being over the measuring range (>300 µIU/mL; n = 3, always the same horse). In 8/61 OSTs with InsMax at T60, 7/46 at T90 and 4/57 at T120, the ID status would have changed from ID to non‐ID if the horse was only sampled at either of the other two timepoints.

### 3.2 Changes throughout the year

**FIGURE 1:**
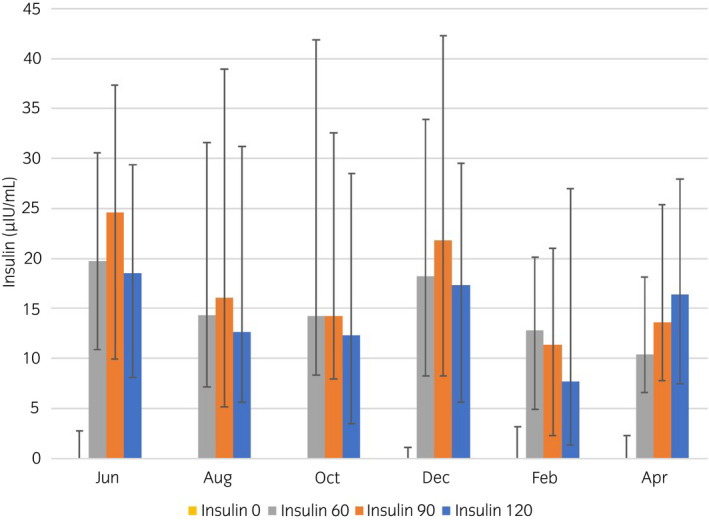
Median (IQ range) serum insulin concentration of 29 horses before (T0) and at 60 (T60), 90 (T90) and 120 (T120) minutes after oral sugar administration. Oral sugar tests were performed every other month for a total of six times

**FIGURE 2:**
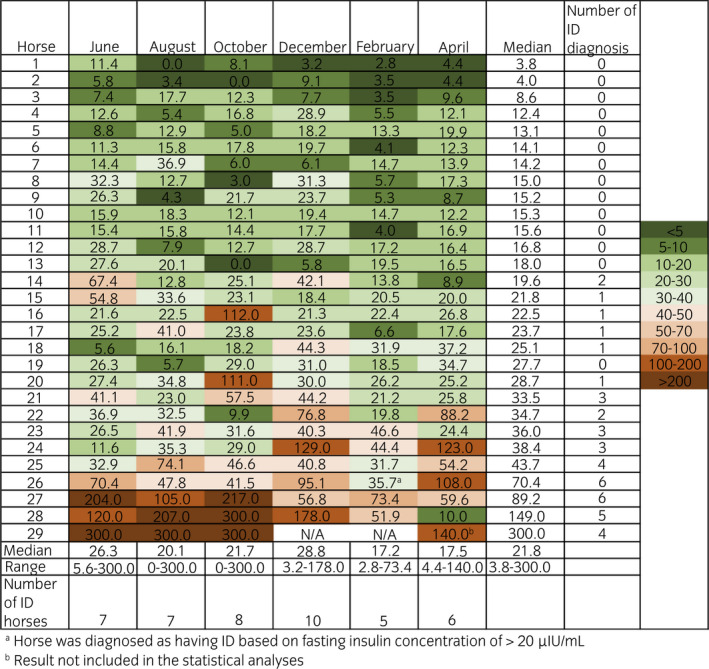
Maximum serum insulin concentration (InsMax, µIU/mL) of 29 horses during oral sugar test performed every other month for six times. Median (range) monthly InsMax of all the horses and the numbers of insulin dysregulation (ID) diagnoses per month and horse are presented. Brown colours indicate ID and green colours normal insulin regulation

**FIGURE 3:**
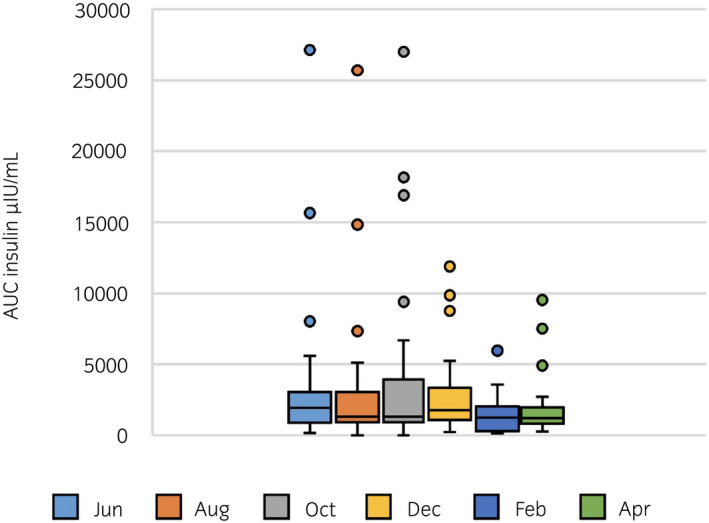
Monthly median (1.5× IQR) area under the curve for serum insulin concentration during oral sugar test (0‐120 min) performed for 29 horses every other month for six times

None of the insulin variables measured during the OST (InsMax, AUC_ins_) had significant variation throughout the year (Figures 1, 2, 3). Three horses were considered to have ID on all sampling dates; however, one of these horses was excluded after the third OST due to acute laminitis. Twelve horses had varying ID status and 14 were classified as non‐ID throughout the study period.

Body condition score was the only variable with significant changes throughout the year (*P* = .04). Compared with December (median 7, range 6‐9), horses in August (median 8, range 6‐9; OR 4.7, 95% CI 1.4‐15.9, *P* = .01) and October (median 8, range 6‐9; OR 6.3, 95% CI 1.8‐21.5, *P* = .004) had increased odds for a higher BCS. Horses in October also had increased odds for a higher BCS compared to February (median 7, range 6‐9; OR 0.25, 95% CI 0.08‐0.86, *P* = .02). No statistically significant changes throughout the year were observed for other variables (Table S1).

Four horses had seasonally increased ACTH values; however, none of these animals had clinical signs consistent with pituitary pars intermedia dysfunction. One of the horses had increased values in June (50.3 pg/mL) and April (36.7 pg/mL), and the other three in April alone (40.2, 35.3, and 40.8 pg/mL). None of these horses were classified as having ID in those months; however, two of these horses had varying insulin status throughout the study period.

The horse that developed clinical laminitis (Obel 1‐2/4) 29 days after the third OST (October) was excluded from subsequent testing due to resultant changes in management (food restriction, stall rest, pain medication). The horse recovered clinically in a month and was given an OST on the final test date in April (results not included in statistical analyses). The InsMax of this individual decreased from >300 µIU/mL (June, August and October) to 140 µIU/mL (5 months after the dietary intervention) and AUC_ins_ from 26 613 µIU/mL (mean of June, August and October) to 9457 µIU/mL. However, adiponectin concentration remained low throughout the study period (median 1.4 µg/mL, range 1.2‐1.6 µg/mL).

### 3.3 Risk factor analysis of InsMax_log_

In the univariate analysis, the following covariate effects on InsMax_log_ were significant: Adipo_log_, age, BCS, CNS, exercise, feeding, and sex (Table S2). None of the interactions of covariate and timepoint were significant, so no interactions were included in the subgroup models. In stage two (subgroup analysis), age (*P* = .002) and sex (*P* = .02) were significant in the demographic model, while Adipo_log_ and CNS were significant (*P* < .001) in the biochemical and phenotypic values model. No variables were significant in the external conditions (feeding/exercise) model. The results of the final model are presented in Table 1 and the relationship between adiponectin and InsMax is presented in Figure S1.

**TABLE 1:** Final results of risk factor analysis for logarithmic transformation of the maximum insulin (InsMax_log_) concentration in oral sugar test (OST). OST was performed on 29 horses every other month for a total of six times

Variable	Category	Estimated increase in InsMax_log_	95% CI^a^	*P*‐value
Sex	Mare versus gelding	0.25	−0.07 to 0.56	.1
Age	1 year	0.08	0.04–0.1	<.001
CNS	1 grade	0.46	0.3–0.7	<.001
Adiponectin_log_	1 log unit (µg/mL)	−0.03	−0.04 to 0.01	<.001

^a^ Confidence interval.

### 3.4 Risk factor analysis of ID

In the univariate analysis, CNS (*P* = .01), exercise (*P* = .05), and scale weight (*P* = .04) were found significant (Table S3). In the subgroup analysis, only exercise remained significant (*P* = .03). The final model showed that the risk of ID was higher in horses with no exercise compared to exercising horses (OR 7.6, 95% CI 1.2‐49.3, *P* = .03).

### 3.5 Group comparisons

Significant differences in adiponectin, neck circumference and height were observed between the two groups (Table 2). The estimated difference in Adipo_log_ between the ID and non‐ID groups was −0.31 (95% CI −0.48 to 0.14, *P* < .001). The neck circumference was estimated to be 2.05 cm (95% CI 0.20‐3.90, *P* = .03) longer and height 2.25 cm (95% CI 0.74‐3.76, *P* = .004) larger in ID horses compared to non‐ID horses.

**TABLE 2:** Group‐wise comparison of selected metabolic and physical variables and age in 29 horses with either insulin dysregulation at least once during the follow‐up period (ID) or never ID (non‐ID) based on an oral sugar test. All variables were measured every other month for a total of six times. Results are presented as the median of medians of the horses of each group (range)

Variable	ID (n = 15)	Non‐ID (n = 14)
Adiponectin (µg/mL)	20.1 (1.5‐46.7)^a^	25.4 (16.1‐41.9)
Glucose (mmol/L)	5.7 (4.8‐6.1)	5.5 (4.8‐6.1)
Widest part of the abdomen (cm)	217.5 (210‐232)	217.0 (203‐228)
Girth (cm)	193.5 (183‐201)	191.5 (182‐203)
Height (cm)	157.0 (149‐168)^a^	155.0 (146‐165)
Neck circumference (cm)	107.5 (97‐116)^a^	104.0 (97‐114)
Scale weight (kg)	572.5 (513‐653)	568.8 (492‐630)
Age (years)	12.0 (7‐19)	13.0 (3‐18)

^a^ Different from non‐ID.

## 4 DISCUSSION

This longitudinal study is the first to evaluate the changes in OST results throughout the year in horses. The OST was performed on 29 horses every other month for a total of six times. None of the insulin variables measured in the OST had significant variation during the study period. The effect of season on the closely related dynamic oral test for ID, oral glucose test, has been previously demonstrated in healthy geldings. In that study, insulin concentration post‐glucose increased from June to September with the highest values in December, indicating that the pancreatic β‐cell sensitivity to glucose may increase when the daylight decreases.^15^ In our study, the highest number of ID horses was also observed in December, although the difference between the other months was not statistically significant. One possible explanation for the different results might relate to geographic locations. The previous study was performed in central Europe (Germany),^15^ whereas our study was conducted in northern Europe (Finland). It has been suggested that geographic location can affect the onset and amplitude of seasonal increase in pituitary hormones. The autumn increase has been shown to occur earlier in northern latitudes, while a greater amplitude in seasonal hormone variation occurs in horses farther south.^28^ Since some pituitary hormones (eg ACTH and α‐MSH) have a role in seasonal regulation of energy metabolism, they may also have an effect on insulin metabolism. However, potential geographical differences in dynamic insulin response have not been described.

Breed differences in insulin sensitivity have been demonstrated,^29^ and Finnhorses may be genetically less predisposed to ID than the Warmblood horses used in the previous study of seasonal variation^15^; and the different breeds included may explain the different findings. The prevalence of ID is quite similar in Finnhorses (16%)^30^ and light breed horses (18%, including Warmblood horses)^31^; however, different methods were used to diagnose ID (OST in Finnhorses and fasting serum insulin in light breed horses) and therefore the results may not be fully comparable.

Although there were no significant annual changes in OST results, the ID status of almost half the horses (12/29) varied throughout the year with no significant peak months. This may be due to changes in management such as feeding or exercise, although these were kept as constant as possible throughout the study period. Horses were handled and fed by the same professionals during the study which probably minimised variation. However, not all changes could be avoided in this field study. Another possible reason for varying results might relate to lack of repeatability in the OST, although it has been shown to be acceptable when binary results were evaluated.^32^, ^33^ However, if those horses with variation in their ID status are true borderline cases, they may be at risk of developing clinical EMS if the environmental conditions alter (eg excess feeding and lack of exercise). Repeated testing may be beneficial for those individuals that are strongly suspected of having EMS based on phenotypic variables.

Age is associated with higher basal serum insulin concentrations^14^, ^34^ and reduced insulin sensitivity in horses.^35^, ^36^ In the current study, increasing age was a significant risk factor for higher InsMax after OST. In humans, increased insulin resistance is also connected with ageing. However, it is not clear if this is a consequence of the biological ageing process or a result of environmental and lifestyle choices.^37^, ^38^

Low serum adiponectin concentrations are associated with diabetes and metabolic syndrome in humans.^39^ In horses, low adiponectin concentrations are associated with laminitis.^27^, ^40^ In our study, the horse that developed laminitis, also had constant hypoadiponectinemia. In one recent study in a herd of ID ponies, no difference in high molecular weight (HMW) adiponectin was observed between laminitic and non‐laminitic ponies. However, in that study laminitis was induced with diet and therefore the risk factors may not have been the same as those in naturally occurring cases.^41^ On the other hand, HMW adiponectin has been shown to correlate negatively with both basal^21^ and postprandial insulin,^42^ and in our study, decreasing adiponectin was a significant risk factor for increased InsMax after OST. Additionally, adiponectin was significantly lower in ID horses vs. non‐ID horses. Therefore, it may be that low adiponectin concentrations are more related to ID than the development of laminitis.

Serum adiponectin concentration did not have significant seasonal variation in the current study. However, seasonality has been shown previously. In one study, up‐regulation of both adiponectin and adiponectin receptor 1 occurred in Finnhorse mares in September (at the end of grazing season) compared to May.^43^ In another study performed on a herd of healthy, Welsh pony mares, plasma adiponectin concentrations showed a progressive decrease from February to October when the ponies were taken from inside to pasture. The BCS increased during the study period and adiponectin concentration was inversely correlated with subcutaneous fat thickness.^16^ In our current study, BCS was also higher after pasture season (August and October) than in December, but this finding did not influence adiponectin. One reason for this may be that the horses in our study were kept on pasture for shorter periods of time (median 1 month) and most were exercising during summer, whereas horses were on pasture the whole summer and did not exercise in previous studies.^16^, ^43^

Body condition score was the only variable with significant changes throughout the year and most likely the higher BCS in August and October versus December and October versus February can be explained by the preceding pasture season. Cresty neck score did not have seasonal variation, but it was a significant risk factor for increasing InsMax. Similar findings have been observed in a previous study where postprandial insulin concentration was positively associated with CNS, and ponies with a CNS ≥3 had five times greater odds of having ID.^42^ Additionally, it was suggested that CNS would be more predictive of ID than BCS. In the present study, CNS and BCS were the same between the ID groups, but ID horses had a longer neck circumference than non‐ID horses. Therefore, it may be, that regional rather than generalised adiposity is more predictive of ID also in Finnhorses.

Regular physical activity has been shown to reduce the risk of insulin resistance even without weight loss in humans.^44^, ^45^ In horses, studies regarding the effect of physical activity on ID have yielded variable results, possibly due to different populations and exercise programmes.^46^, ^47^, ^48^, ^49^, ^50^ In the present study, horses that did not exercise had an almost eightfold higher risk for ID than the horses that did exercise. However, the amount and intensity of exercise were not included in statistical analyses due to missing values and inaccuracies in data. The horses that did exercise, exercised fairly lightly but regularly (mean 1.2 hours riding or driving school lessons per day), which appeared to be sufficient to reduce the risk of ID in this population.

The maximum postprandial insulin concentration was noticed at T120 in over a third (57/171) of OSTs performed. Therefore, the generally used time interval at T60‐90 for sampling may not always be enough to catch the insulin peak. However, only in 4/57 OSTs the ID status would have changed, if the horse was only sampled at T60 or T90. Ideally, based on the current study, more than one postprandial sample should be taken to get more accurate results compared to only sampling once between T60 and T90. Another interesting option could be to use AUC_ins_ for diagnosing ID; however, there are no published cut‐off values for AUC_ins_. In addition, it remains unknown whether the peak (if cleared quickly) or the shape (AUC) of the postprandial insulin curve is more substantial for the development of laminitis.

One of the limitations of this study is that the study population was a convenience sample: We selected all the available animals of the two institutes that fulfilled the inclusion criteria. Power calculations were not performed; however, in the previous equine studies regarding seasonality of insulin, the population size has been quite similar to the current study.^4^, ^5^, ^7^, ^13^, ^14^, ^15^, ^16^, ^17^, ^18^ Another limitation is that the population included only Finnhorses. Therefore, the results may only be pertinent to this breed.

## 5 CONCLUSIONS

Insulin dysregulation status can vary throughout the year in some horses. However, this does not seem to follow any seasonal pattern. Repeated testing of phenotypically suspicious animals may, therefore, be beneficial. Lack of exercise markedly increased the risk of ID; however, further studies to determine the amount and intensity of exercise, are warranted.

## CONFLICT OF INTERESTS

No competing interests have been declared.

## AUTHOR CONTRIBUTIONS

N. Karikoski, J. Box and M. Raekallio contributed to study design, study execution, data analysis and interpretation, preparation of the manuscript and final approval of the manuscript. A. Mykkänen and V. Kotiranta contributed to study execution, preparation of the manuscript and final approval of the manuscript. N. Karikoski, J. Box and M. Raekallio had full access to all the data and take responsibility for the integrity of the data and the accuracy of the data analysis.

## ETHICAL ANIMAL RESEARCH

The study protocol was approved by the National Animal Experimentation Board of Finland (ESAVI/9250/2018).

## INFORMED CONSENT

Owners gave consent for their animals' inclusion in the study.

### PEER REVIEW

The peer review history for this article is available at https://publons.com/publon/10.1111/evj.13529.

## Supporting information

Fig S1

Table S1

Table S2

Table S3

German Summary

## Data Availability

The data that support the findings of this study are available from the corresponding author upon reasonable request.
