# Supplementary material for: Variation in insulin response to oral sugar test in a cohort of horses throughout the year and evaluation of risk factors for insulin dysregulation
Source: Equine Vet J. 2021 Nov 8;54(5):905–13. doi: 10.1111/evj.13529 (PMC9545906; doi:10.1111/evj.13529)
Supplement: Supplementary file 1 — Fig S1 [file EVJ-54-905-s001.pdf]

Equine Veterinary Journal BEVA

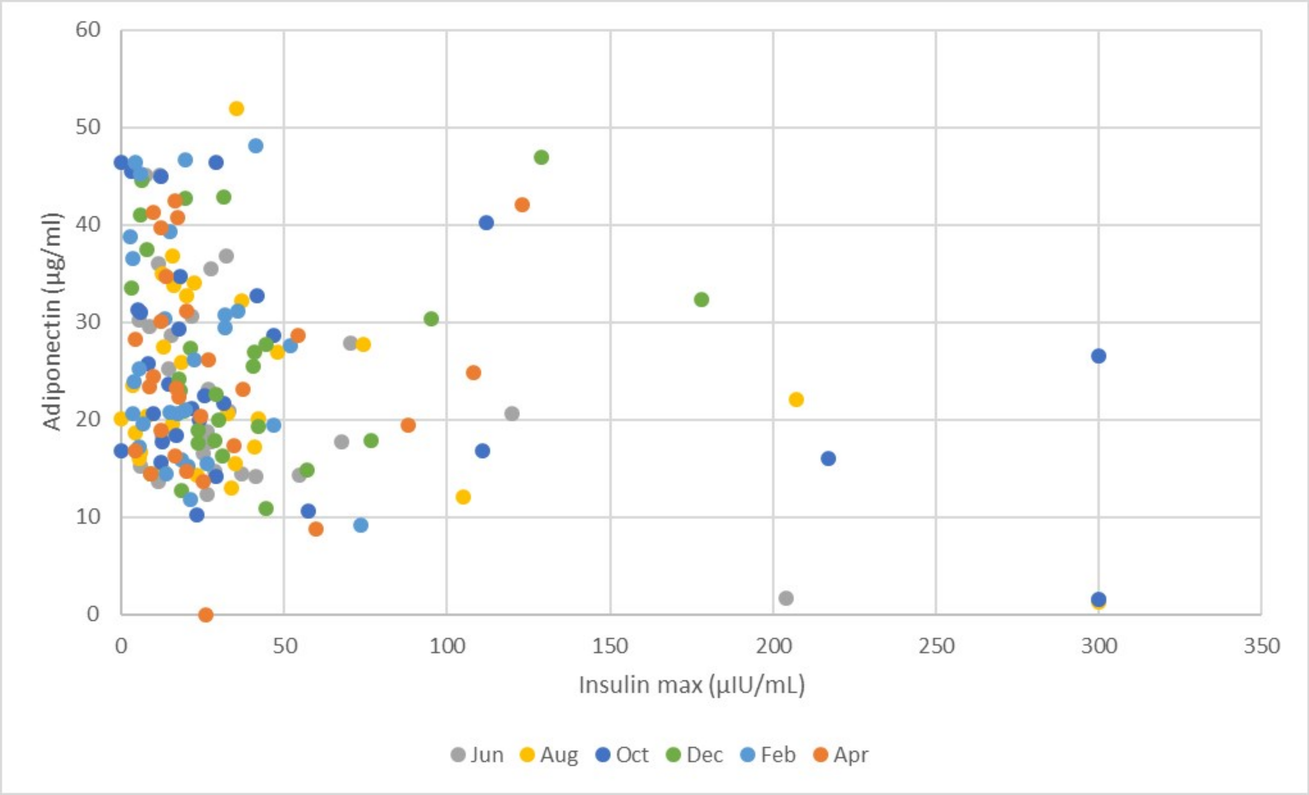

Figure S1: Scatter plot showing the relationship between serum adiponectin concentration and maximum serum insulin concentration (0, 60, 90, or 120 minutes) during oral sugar test (OST). Adiponectin was measured and OST performed on 29 horses every other month for a total of six times.
