# Supplementary material for: Variation in insulin response to oral sugar test in a cohort of horses throughout the year and evaluation of risk factors for insulin dysregulation
Source: Equine Vet J. 2021 Nov 8;54(5):905–13. doi: 10.1111/evj.13529 (PMC9545906; doi:10.1111/evj.13529)
Supplement: Supplementary file 2 — Table S1 [file EVJ-54-905-s005.pdf]

Equine Veterinary Journal BEVA

Table S1: Physical and biochemical variables (median and range) of 29 horses collected every other month for a total of six times.

Variable	June	August	October	December	February	April
Scale weight (kg)	578 (509–664)	573 (480–668)	565 (484–652)	570 (499–649)	568 (507–657)	568 (485–675)
Heart girth (cm)	194 (181–202)	191 (179–202)	192 (182–206)	194 (183–206)	193 (183–201)	192 (182–203)
Widest part of the abdomen (cm)	218 (204–231)	215 (205–236)	219 (207–234)	220 (209–234)	218 (210–233)	217 (205–230)
Cresty neck score (0–5)	3 (1–4)	3 (2–4)	3 (2–4)	3 (2–4)	3 (2–4)	3 (2–4)
Neck circumference (cm)	111 (96–120)	108 (95–115)	109 (96–116)	104 (94–114)	105 (91–113)	106 (95–116)
Adiponectin (µg/ml)	18.8 (1.6–45.1)	20.7 (1.3–52.0)	22.5 (1.5–46.4)	23.6 (10.9–47.0)	24.6 (9.2–48.2)	23.4 (8.8–42.5)
Fasting glucose (mmol/l)	5.6 (4.6–7.9)	5.2 (3.8–6.6)	5.2 (4.2–6.5)	5.6 (4.7–7.0)	5.7 (3.4–6.7)	5.7 (3.3–6.9)
Triglycerides (mmol/l)	0.3 (0.1–1.0)	0.3 (0.1–0.9)	0.3 (0.1–0.8)	0.3 (0.2–0.4)	0.2 (0.1–0.4)	0.3 (0.2–0.5)
ACTH (pg/ml)	20.6 (14.1–50.3)	25.0 (14.1–58.7)	33.1 (23.6–75.8)	20.2 (13.6–34.4)	21.2 (15.1–32.2)	25.6 (18.9–40.8)
