# Supplementary material for: Variation in insulin response to oral sugar test in a cohort of horses throughout the year and evaluation of risk factors for insulin dysregulation
Source: Equine Vet J. 2021 Nov 8;54(5):905–13. doi: 10.1111/evj.13529 (PMC9545906; doi:10.1111/evj.13529)
Supplement: Supplementary file 3 — Table S2 [file EVJ-54-905-s004.pdf]

Equine Veterinary Journal BEVA

Table S2: Univariable analysis of the effects of potential risk factors for maximum serum insulin (log) concentration (0, 60, 90 or 120 minutes) during oral sugar test (OST). OST was performed on 29 horses every other month for a total of six times.

**Variable**	**Estimate (log)**	**95% CI[a]**	**p–value**
Gender (mare vs. gelding)	0.468	0.133; 0.803	.007
Age (1 year)	0.089	0.042; 0.135	< .001
Exercise (exercise vs. no exercise)	-0.942	-1.271; -0.613	< .001
Feeding (pasture vs. inside)	0.753	0.209; 1.297	.007
Scale weight (1 kg)	0.003	-0.001; 0.007	.1
$Adiponectin_{log}$ (1 log unit)	-0.024	-0.039; -0.009	.002
Cresty neck score (1 unit)	0.519	0.302; 0.736	< .001
Neck circumference (1 cm)	0.027	-0.000; 0.054	.05
Widest part of the abdomen (1 cm)	0.017	-0.008; 0.042	.1
Heart-girth (1 cm)	-0.001	-0.029; 0.027	> .9
Body condition score (1 unit)	0.225	0.049; 0.401	.01

[a] Confidence interval
