# Supplementary material for: Variation in insulin response to oral sugar test in a cohort of horses throughout the year and evaluation of risk factors for insulin dysregulation
Source: Equine Vet J. 2021 Nov 8;54(5):905–13. doi: 10.1111/evj.13529 (PMC9545906; doi:10.1111/evj.13529)
Supplement: Supplementary file 4 — Table S3 [file EVJ-54-905-s003.pdf]

Table S3: Univariable analysis of the effects of potential risk factors for insulin dysregulation (ID). ID was diagnosed if insulin concentration was ≥ 20 μIU/ml at T0 or ≥ 40 μIU/ml at either T60, T90, or T120 or all timepoints after oral sugar administration (OST). OST was performed on 29 horses every other month for a total of six times.

**Variable**	**Odds ratio**	**95% CI[a]**	**p–value**
Gender (mare vs. gelding)	3.02	0.404; 22.617	0.2
Age (1 year)	1.27	0.946; 1.709	0.1
Exercise (exercise vs. no exercise)	0.14	0.018; 1.000	0.05
Feeding (pasture vs. inside)	3.83	0.516; 28.478	0.1
Scale weight (1 kg)	1.02	1.000; 1.046	0.04
$Adiponectin_{log}$ (1 unit)	0.94	0.862; 1.031	0.1
Cresty neck score (1 unit)	3.64	1.331; 9.943	0.01
Neck circumference (1 cm)	1.13	0.981; 1.312	0.08
Widest part of the abdomen (1 cm)	1.05	0.936; 1.172	0.4
Heart-girth (1 cm)	1.06	0.921; 1.211	0.4
Body condition score (1 unit)	1.47	0.693; 3.130	0.3

[a] Confidence interval
