# Supplementary material for: Variation in insulin response to oral sugar test in a cohort of horses throughout the year and evaluation of risk factors for insulin dysregulation
Source: Equine Vet J. 2021 Nov 8;54(5):905–13. doi: 10.1111/evj.13529 (PMC9545906; doi:10.1111/evj.13529)
Supplement: Supplementary file 5 — German Summary [file EVJ-54-905-s002.docx]

**Variation der Insulinantwort nach oralem Zuckertest in einer Kohorte von Pferden im Verlauf eines Jahres und Evaluation von Risikofaktoren für Insulindysregulation**

Ninja P. Karikoski^*^, Justin R. Box, Anna K. Mykkänen, Veikko V. Kotiranta and Marja R. Raekallio

Department of Equine and Small Animal Sciences, Faculty of Veterinary Medicine, University of Helsinki, Finland.

*E-Mail-Adresse des korrespondierenden Autors**:** [ninja.karikoski@helsinki.fi](mailto:ninja.karikoski@helsinki.fi)

**Schlüsselwörter:** Pferd, saisonal, Insulindysregulation, endokrin

**Kopfzeile:** Variation des OST bei Pferden und Risikofaktoren für ID

**Zusammenfassung**

**Hintergrund:** Der orale Zuckertest (Oral Sugar Test, OST) wird häufig zur Diagnose von Insulindysregulation (ID) und Equinen Metabolischen Syndrom (EMS) verwendet. Mögliche saisonale Abweichungen der OST Ergebnisse wurden bisher allerdings nicht untersucht.

**Ziel der Studie:** Ermittlung einer möglichen Abweichung der Insulinantwort nach OST im Verlauf eines Jahres und mit maximaler Insulinkonzentration (InsMax) und ID assoziierte Risikofaktoren.

**Studiendesign:** Prospektive Kohorten-Längsschnittstudie

**Methodik:** Der OST wurde sechs Mal (alle zwei Monate) bei 29 Finnpferden durchgeführt. Totale Adiponektinkonzentrationen und phenotypische Variablen in Verbindung mit Adipositas wurden zusätzlich gemessen. Abweichungen von InsMax, Adiponektin, Körpergewicht, Body-Condition-Score, Score der Fettdepots am Mähnenkamm und Glukosekonzentration nach dem Fasten wurden bewertet. Eine Analyse der Risikofaktoren für InsMax und ID status wurde durchgeführt, und die ID Gruppen miteinander verglichen.

**Ergebnisse:** Vierzehn Pferde wurden durchgehend als nicht-ID kategorisiert und 15 zeigten ID mindestens ein Mal in der Beobachtungsperiode. Der ID-Status von 12 Pferden variierte während des Jahres, jedoch zeigten weder Insulinvariablen gemessen während des OST noch Adiponektin signifikante saisonale Abweichungen. Steigendes Alter, Score der Fettdepots am Mähnenkamm und abnehmendes Adiponektin waren Risikofaktoren für eine hohe InsMax nach OST. Das Risiko für ID war höher bei Pferden ohne Bewegung verglichen mit Pferden, die bewegt wurden (OR 7.6, 95% CI 1.2-49.3, p=0.03). Pferde mit ID hatten niedrigere Serumkonzentrationen von Adiponektin, einen größeren halsumfang und waren größer als Pferde in der nicht-ID Gruppe.

**Haupteinschränkungen:** Die Umweltbedingungen (Fütterung, Bewegung) waren nicht konstant für alle Pferde im Verlauf der Studie und lediglich eine Rasse wurde verwendet.

**Schlussfolgerungen:** Weder OST noch Adiponektin variiert mit der Jahreszeit; jedoch gab es eine substanzielle Anzahl an Pferden mit variablem ID Status im Verlauf des Jahres, für welche wiederholte OSTs vorteilhaft sein könnten. Mangel an Bewegung war ein Risikofaktor für ID.
